# Supplementary material for: Genetically Engineered MRI-Trackable Extracellular Vesicles as SARS-CoV-2 Mimetics for Mapping ACE2 Binding In Vivo
Source: ACS Nano. 2022 Aug 3;16(8):12276–89. doi: 10.1021/acsnano.2c03119 (PMC9364977; doi:10.1021/acsnano.2c03119)
Supplement: Supplementary file 1 — nn2c03119_si_001.pdf [file nn2c03119_si_001.pdf]

Supplementary Information

Genetically engineered MRI-trackable extracellular vesicles as SARS-CoV-2 mimetics for mapping ACE2 binding *in vivo*

Andrea Galisova[1], Jiri Zahradnik[2], Hyla Allouche-Arnon[1], Mattia I. Morandi[2], Paula Abou Karam[2], Michal Fisler[1], Ori Avinoam[2], Neta Regev-Rudzki[2], Gideon Schreiber[2], Amnon Bar-Shir[1*]

[1]Department of Molecular Chemistry and Materials Science, [2]Department of Biomolecular Sciences, Weizmann Institute of Science, Rehovot, 7610001, Israel

*Corresponding Author: amnon.barshir@weizmann.ac.il

Supplementary Figures

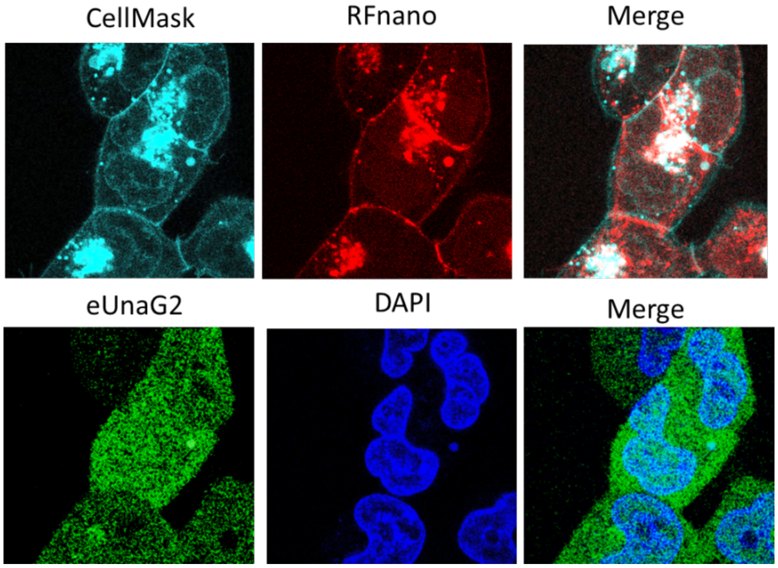

Figure S1. Confocal microscopy of RBD cells. A cell mask to label cell membranes was co-localized with RFnano fused with RBD, confirming membrane expression of RBD. The eUnaG2 protein used for FACS sorting and establishment of a stable cell line was localized in cytoplasm.

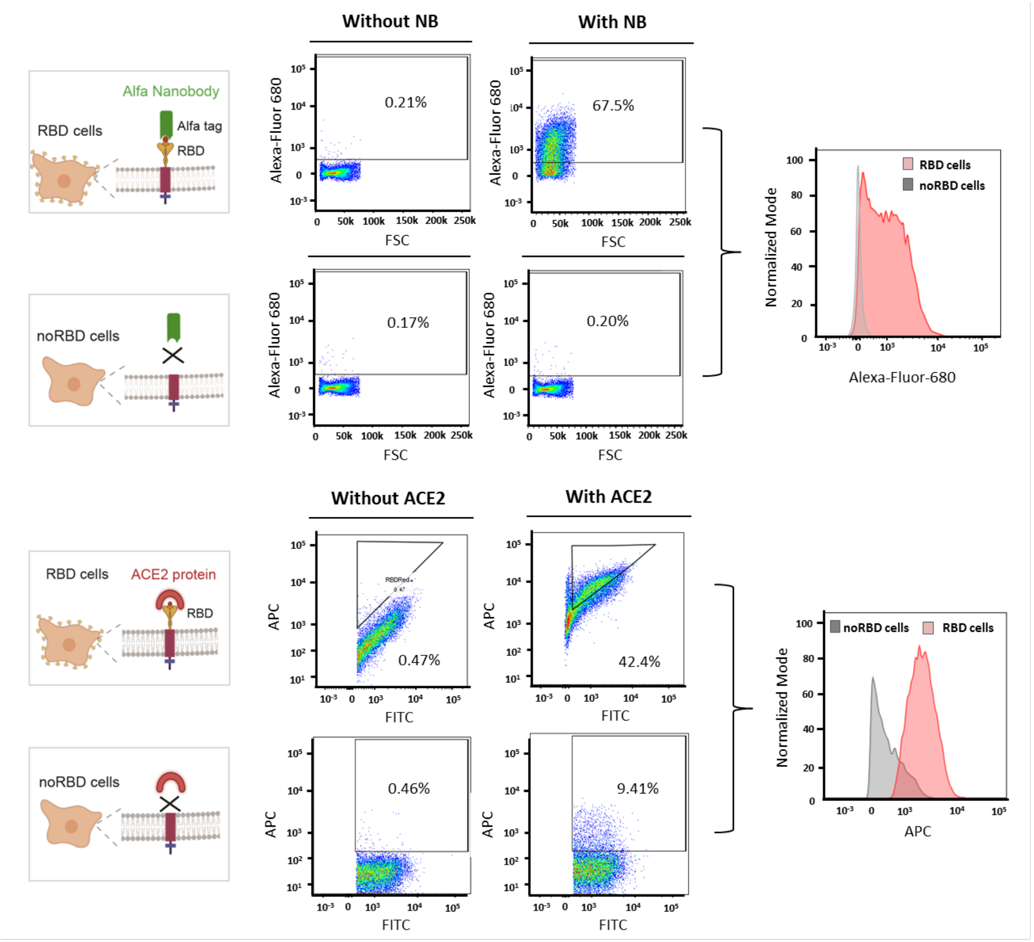

Figure S2. Detection of ALFA tag (upper panel) and binding to ACE2 protein (bottom panel) in parental RBD/noRBD cells by flow cytometry. Gating strategies for FACS analysis and histograms depicting the differences between RBD and noRBD cells.

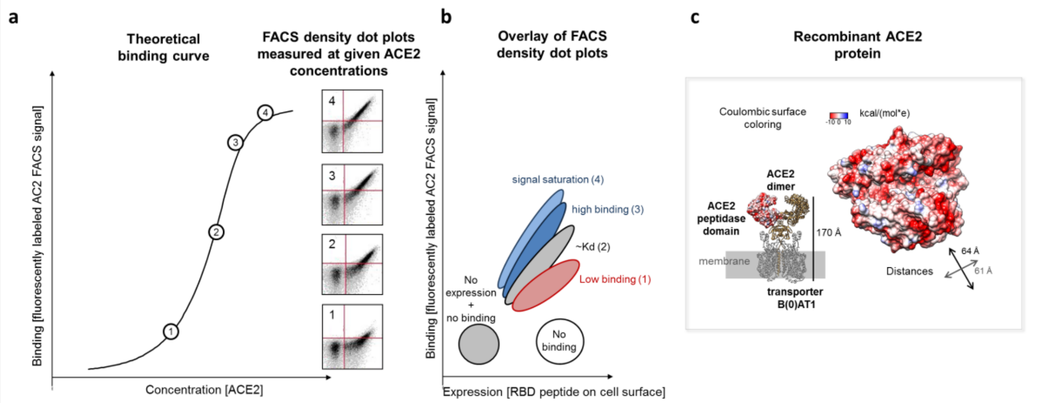

Figure S3. Binding assay and affinity curve determination using FACS. The binding constant of the RBD expressed on the cell surface to ACE2 was determined by incubation of the examined cells with ten different concentrations of fluorescently labeled ACE2 protein and a subsequent analysis by FACS. The theoretical binding curve is depicted in **(a)** with four FACS representative density dot plots measured at four different concentrations (insets, 1-4). Schematic overlay of the FACS signal contours of cells incubated with different ACE2 concentrations. **(b)** Differences in the binding signals can be analyzed by changes in binding signal intensities (y-axis) and the skewness of the FACS signal given by the RBD expression. **(c)** The Coulombic surface coloring map of the recombinant ACE2 protein used in the assay.

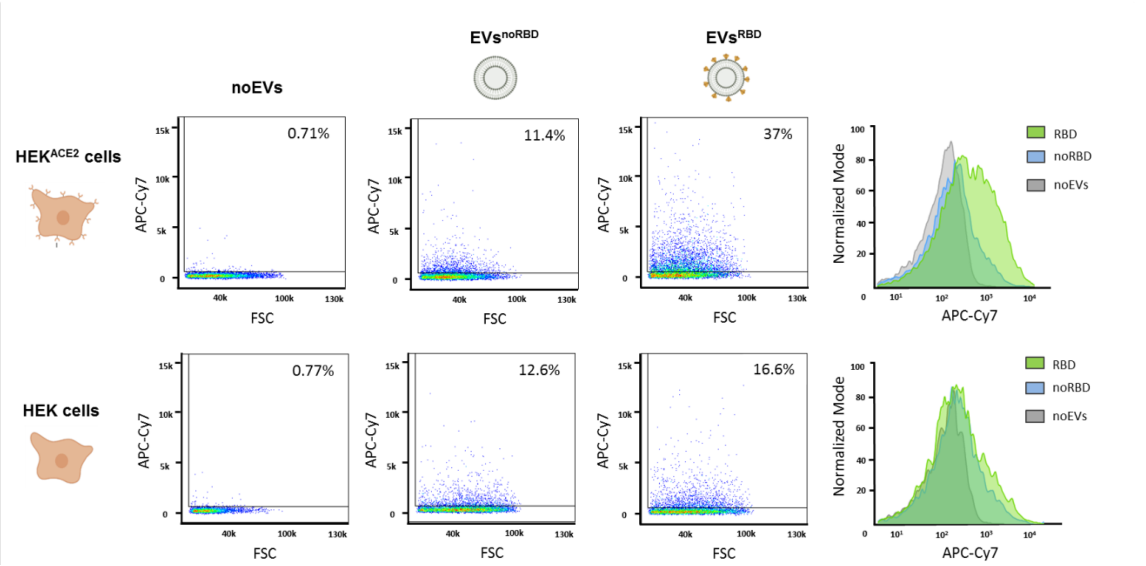

Figure S4. Targeted uptake of EVs in cells. Flow cytometry analysis of DiR-labeled EVs^{RBD} and EVs^{noRBD} accumulated in HEK (control) and ACE2-expressing cells—FACS data shown as dot plots (left) and as histograms (right) for comparison. The fluorescent signals of HEK and HEK^{ACE} cells incubated without EVs are shown to emphasize the natural uptake of HEK-derived EVs in HEK cells.

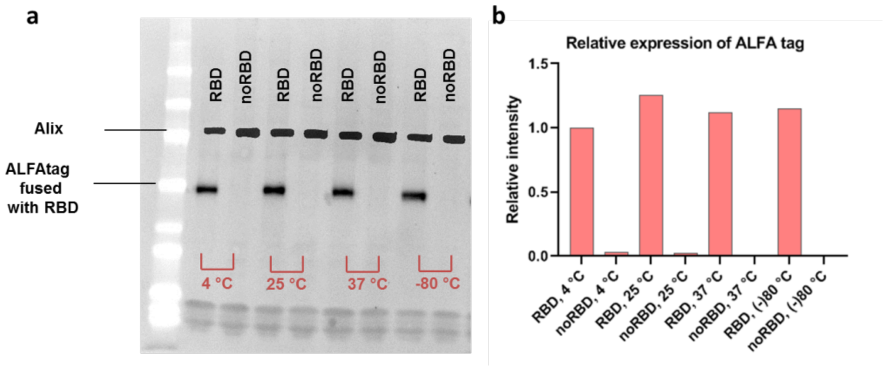

Figure S5: Stability of the formulations. **(a)** Western blot membrane containing proteins expressed in EVs^{RBD} and EVs^{noRBD}– housekeeping protein Alix and targeting protein RBD fused with ALFA tag. **(b)** Relative expression of the targeting peptide normalized to Alix.

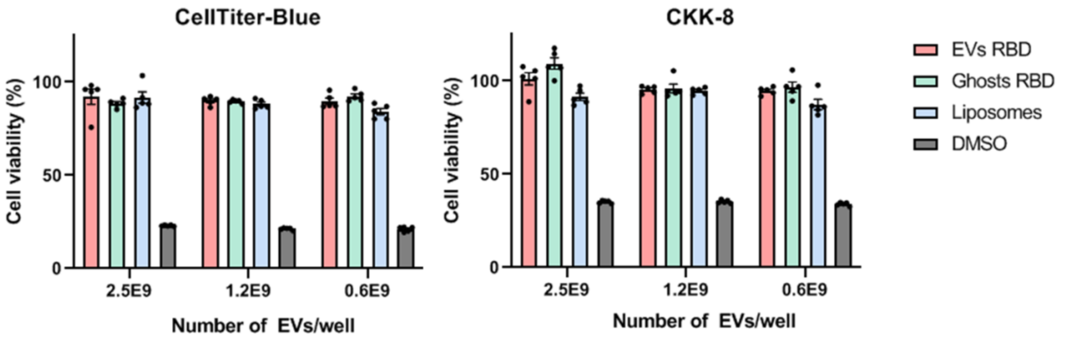

Figure S6. Toxicity evaluation of cells with incubated nanoformulations. CellTiter-Blue and CKK-8 assay of HEK293T cells incubated with three vesicle formulations – EVs, vesicle ghosts, and liposomes for four hours.

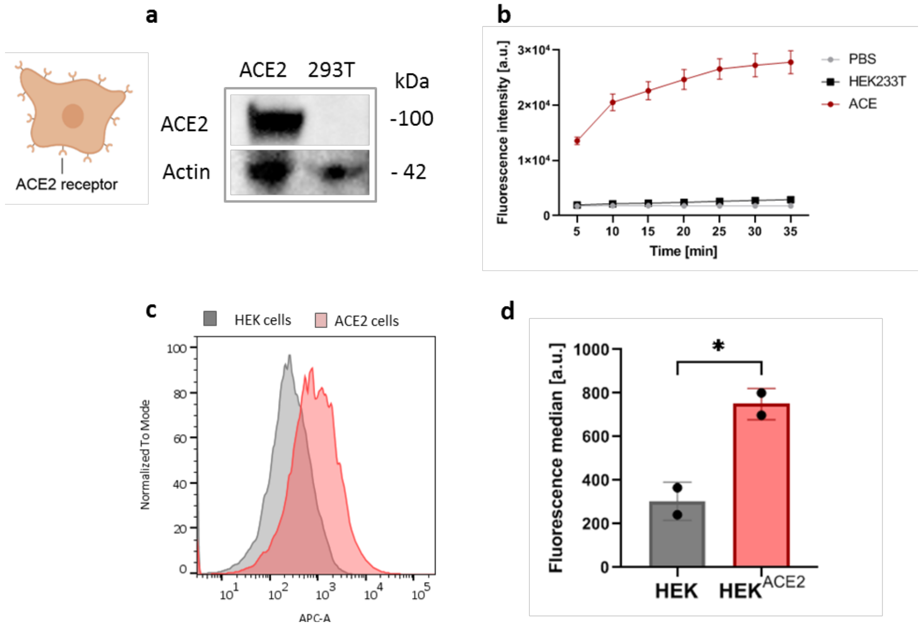

Figure S7. Confirmation of ACE2 in ACE2-expressing stable cell line. (a) Western blot, **(b)** enzymatic detection and FACS analysis of HEK and HEK^{ACE2} cells shown as **(c)** histogram and **(d)** quantification of fluorescence median.

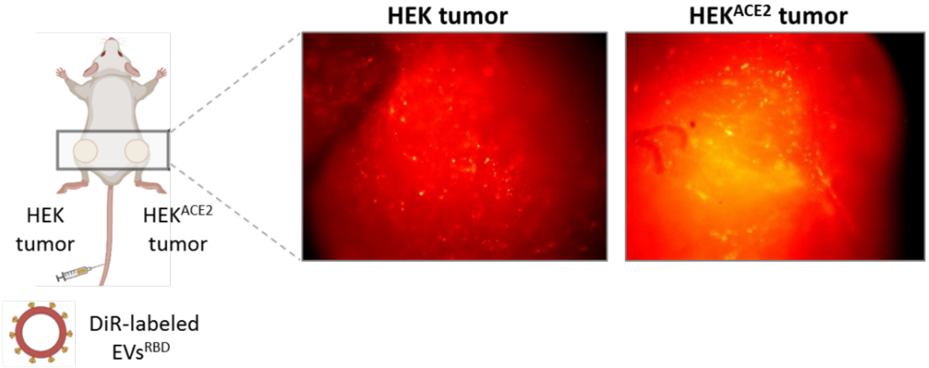

Figure S8. Accumulation of EVs^{RBD} in mice. Intravital microscopy of tumor tissue showing individual EVs accumulated in the tumors.

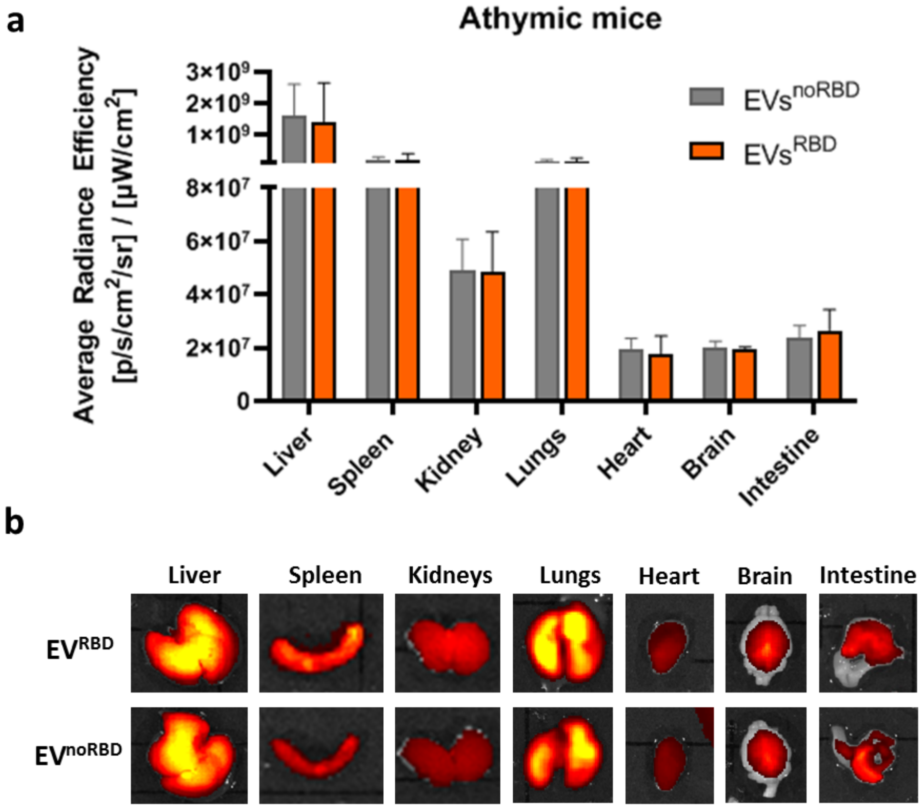

Figure S9. Biodistribution of EVs^{noRBD} and EVs^{RBD} in athymic Foxnunu mice with induced tumors. (a) Quantification of the fluorescent signals and **(b)** representative fluorescent images of organs.

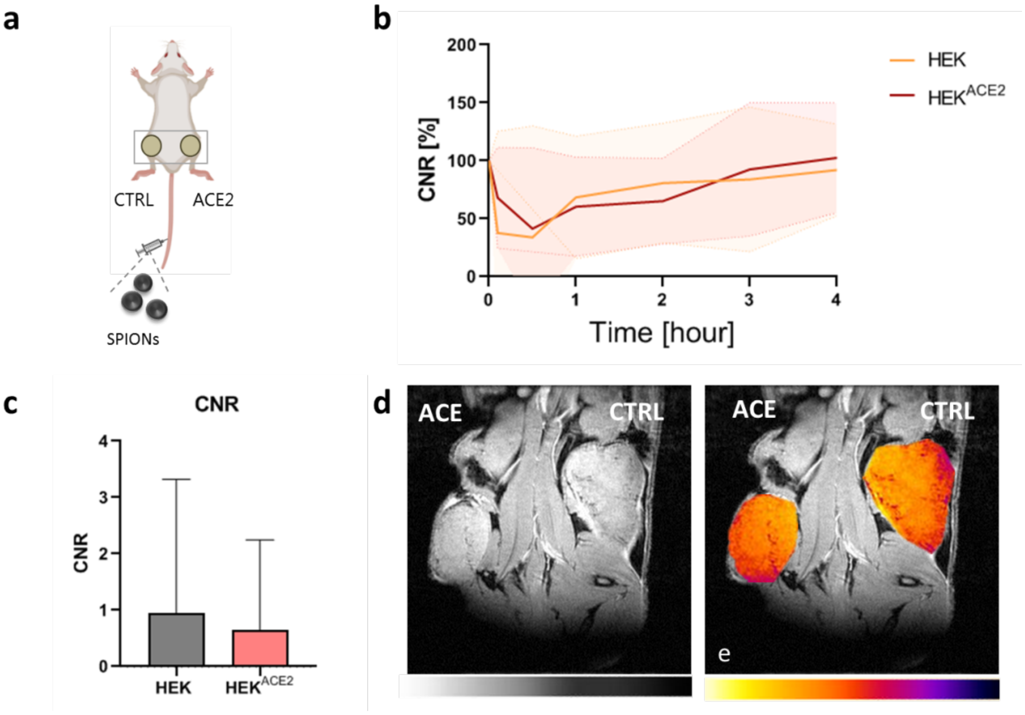

Figure S10. MRI of mice after injection of SPIONs. (a) Schematic illustration of the experiment. **(b)** Time course of the MR signals from HEK and HEK^{ACE} tumors after the injection of SPIONs solution intravenously. **(c)** Calculated contrast-to-noise ratios (CNR) of tumors four hours after SPIONs injection. Representative images of tumors: ACE and control (CTRL) after SPIONs injection – a T2*-weighted image **(d)** and a color-coded image **(e)**.

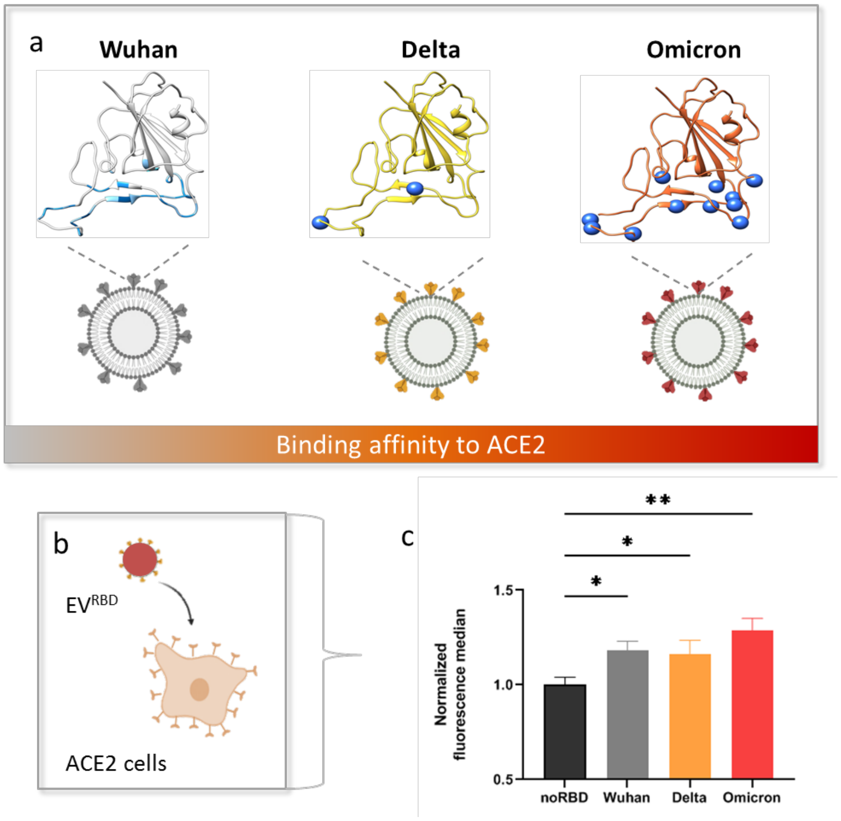

Figure S11: EVs representation of SARS-CoV-2 mutants. (a) Location of the RBD domain mutations characteristic for the Wuhan, Delta and Omicron variants of SARS-CoV-2 depicted in the Wuhan structure (pdb 6m17:f); the variations from the Wuhan variant are shown as blue circles. **(b)** Schematic illustration of the experiment depicting the binding of EVsRBD to ACE2 expressing at the surface of cells. **(c)** Flow cytometry analysis of ACE2-expressing cells (n=3) incubated with fluorescently labeled EVs: EVs^{noRBD}, EVs$^{RBD\text{-}Wuhan}$, EVs$^{RBD\text{-}Delta}$ or EVs$^{RBD\text{-}Omicron}$. Data are presented as mean values ± s.d. Statistics: two-tailed unpaired Student's t-test with *p-value<0.05 and **p-value<0.01.

Supplementary sequences:

RBD-62
ACCAACCTGTGCCCGTTTGGCGAAGTGTTTAACGCGACCCGCTTTGCGAGCGTGTATGCGTGG
AACCGCAAACGCTTTAGCAACTGCGTGGCGGATTATAGCGTGCTGTATAACAGCGCGAGCTTT
AGCACCTTTAAATGCTATGGCGTGAGCCCGACCAAACTGAACGATCTGTGCTTTACCAACGTG
TATGCGGATAGCTTTGTGATTCGCGGCGATGAAGTGCGCCAGATTGCGCCGGGCCAGACCGGC
AAAATTGCGGATTATAACTATAAACTGCCGGATGATTTTACCGGCTGCGTGATTGCGTGGAAC
AGCAACAACCTGGATAGCAAAAAAGGCGGCAACTATAACTATCTGTATCGCCTGTTTCGCAA
AAGCAAACTGAAACCGTTTGAACGCGATACCAGCATGGAAATTTATCAGGCGGGCAACACCC
CGTGCAACGGCGTGAAAGGCTTTAACTGCTATTTTCCGCTGCAGAGCTATGGCTTTCGCCCGA
CCTATGGCGTGGGCTATCAGCCGTATCGCGTGGTGGTGCTGAGCTTTGAACTGCTGCATGCGC
CGGCGACCGTGTGCGGCCCGAAA

RBD-Wuhan
TGCCCTTTTGGTGAAGTTTTTAACGCCACCAGGTTTGCCTCTGTCTATGCCTGGAACAGGAAGA
GGATTAGCAACTGTGTGGCTGACTACTCTGTGCTCTACAACTCTGCCTCCTTCAGCACCTTCAA
GTGTTATGGAGTGAGCCCAACCAAACTGAATGACCTGTGTTTCACCAATGTCTATGCTGACTC
CTTTGTGATTAGGGGAGATGAGGTGAGACAGATTGCCCCTGGACAAACAGGCAAGATTGCTG
ACTACAACTACAAACTGCCTGATGACTTCACAGGCTGTGTGATTGCCTGGAACAGCAACAACC
TGGACAGCAAGGTGGGAGGCAACTACAACTACCTCTACAGACTGTTCAGGAAGAGCAACCTG
AAACCATTTGAGAGGGACATCAGCACAGAGATTTACCAGGCTGGCAGCACACCATGTAATGG
AGTGGAGGGCTTCAACTGTTACTTTCCACTCCAATCCTATGGCTTCCAACCAACCAATGGAGT
GGGCTACCAACCATACAGGGTGGTGGTGCTGTCCTTTGAACTGCTCCATGCCCCTGCCACAGT
GTGTGGACCAAAG

RBD-Delta
TGCCCTTTTGGTGAAGTTTTTAACGCCACCAGGTTTGCCTCTGTCTATGCCTGGAACAGGAAGA
GGATTAGCAACTGTGTGGCTGACTACTCTGTGCTCTACAACTCTGCCTCCTTCAGCACCTTCAA
GTGTTATGGAGTGAGCCCAACCAAACTGAATGACCTGTGTTTCACCAATGTCTATGCTGACTC
CTTTGTGATTAGGGGAGATGAGGTGAGACAGATTGCCCCTGGACAAACAGGCAAGATTGCTG
ACTACAACTACAAACTGCCTGATGACTTCACAGGCTGTGTGATTGCCTGGAACAGCAACAACC
TGGACAGCAAGGTGGGAGGCAACTACAACTACAGATACAGACTGTTCAGGAAGAGCAACCTG
AAACCATTTGAGAGGGACATCAGCACAGAGATTTACCAGGCTGGCAGCAAACCATGTAATGG
AGTGGAGGGCTTCAACTGTTACTTTCCACTCCAATCCTATGGCTTCCAACCAACCAATGGAGT
GGGCTACCAACCATACAGGGTGGTGGTGCTGTCCTTTGAACTGCTCCATGCCCCTGCCACAGT
GTGTGGACCAAAG

RBD-Omicron (version_3S)
TGCCCTTTTGGTGAAGTTTTTAACGCCACCAGGTTTGCCTCTGTCTATGCCTGGAACAGGAAGA
GGATTAGCAACTGTGTGGCTGACTACTCTGTGCTCTACAACTCTGCCTCCTTCAGCACCTTCAA
GTGTTATGGAGTGAGCCCAACCAAACTGAATGACCTGTGTTTCACCAATGTCTATGCTGACTC
CTTTGTGATTAGGGGAGATGAGGTGAGACAGATTGCCCCTGGACAAACAGGCAATATTGCTG
ACTACAACTACAAACTGCCTGATGACTTCACAGGCTGTGTGATTGCCTGGAACAGCAACAAAC
TGGACAGCAAGGTGTCTGGCAACTACAACTACCTCTACAGACTGTTCAGGAAGAGCAACCTG
AAACCATTTGAGAGGGACATCAGCACAGAGATTTACCAGGCTGGCAACAAACCATGTAATGG
AGTGGCCGGCTTCAACTGTTACTTTCCACTCAAGTCCTATTCTTTCCGTCCAACCTACGGAGTG
GGCCATCAACCATACAGGGTGGTGGTGCTGTCCTTTGAACTGCTCCATGCCCCTGCCACAGTG
TGTGGACCAAAG

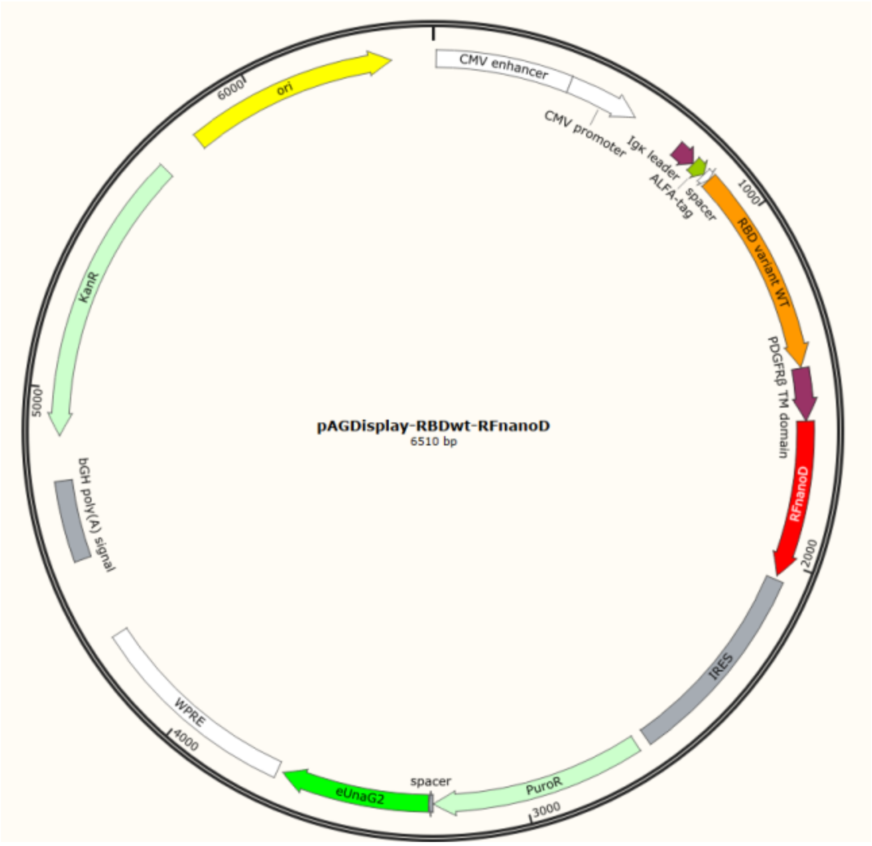

Supplementary plasmid map. A map of the pAGDisplay-RBD plasmid.
